# New groups of highly divergent proteins in families as old as cellular life with important biological functions in the ocean

**DOI:** 10.1186/s40793-025-00697-3

**Published:** 2025-06-11

**Authors:** Duncan Sussfeld, Romain Lannes, Eduardo Corel, Guillaume Bernard, Pierre Martin, Eric Bapteste, Eric Pelletier, Philippe Lopez

**Affiliations:** 1 Institut de Systématique, Evolution, Bioaffiliationersité (ISYEB), Sorbonne Université, CNRS, Museum National d’Histoire Naturelle, EPHE, Université des Antilles, Paris, France; 2 Génomique Métabolique, Genoscope, Institut François-Jacob, CEA, CNRS, Université d’Evry, Université Paris-Saclay, Evry, 91000 France; 3 Research Federation for the Study of Global Ocean Systems Ecology and Evolution, FR2022/Tara Oceans GOSEE, Paris, 75016 France

**Keywords:** Microbial dark matter, Sequence similarity networks, Distant homology, Microbiome

## Abstract

**Background:**

Metagenomics has considerably broadened our knowledge of microbial diversity, unravelling fascinating adaptations and characterising multiple novel major taxonomic groups, e.g. CPR bacteria, DPANN and Asgard archaea, and novel viruses. Such findings profoundly reshaped the structure of the known Tree of Life and emphasised the central role of investigating uncultured organisms. However, despite significant progresses, a large portion of proteins predicted from metagenomes remain today unannotated, both taxonomically and functionally, across many biomes and in particular in oceanic waters.

**Results:**

Here, we used an iterative, network-based approach for remote homology detection, to probe a dataset of 40 million ORFs predicted in marine environments. We assessed the environmental diversity of 53 core gene families broadly distributed across the Tree of Life, with essential functions including translational, replication and trafficking processes. For nearly half of them, we identified clusters of remote environmental homologues that showed divergence from the known genetic diversity comparable to the divergence between Archaea and Bacteria, with representatives distributed across all the oceans. In particular, we report the detection of environmental clades with new structural variants of essential SMC (Structural Maintenance of Chromosomes) genes, divergent polymerase subunits forming deep-branching clades in the polymerase tree, and variant DNA recombinases in Bacteria as well as viruses.

**Conclusions:**

These results indicate that significant environmental diversity may yet be unravelled even in strongly conserved gene families. Protein sequence similarity network approaches, in particular, appear well-suited to highlight potential sources of biological novelty and make better sense of microbial dark matter across taxonomical scales.

**Supplementary Information:**

The online version contains supplementary material available at 10.1186/s40793-025-00697-3.

## Background

Over the last decades, novel sequencing methods have allowed microbiologists to appreciate the ubiquity and abundance of uncultured organisms [1–5], and access microorganisms’ genomes beyond the isolation-cultivation dogma issued from the Koch principles [6] that underpinned microbiological studies for decades. Metagenomic studies [7] have led to an unprecedented broadening of our knowledge of microbial diversity [8], from the unravelling of microbial adaptations and interactions in numerous environments [9–12] to the characterisation of multiple novel major taxonomic groups [13–17] – most notably CPR bacteria [13, 18, 19], DPANN archaea [18, 20, 21] and Asgard archaea [22–24], profoundly reshaping the structure of the Tree of Life. Large groups of novel viruses [25–27] and mobile elements [28] have also been unearthed. Together, these major discoveries emphasise the central role of investigating yet uncultured organisms, believed to constitute the majority of overall microbial lineages [3, 29], in addressing many fundamental questions of biology and evolutionary biology.

Over time, as cultivation-independent sequencing efforts are carried out in an increasing range of ecosystems, discovery events of novel branches near the base of the tree of life are predicted to become less frequent [8, 17]. For instance, an extensive study of over 50,000 MAGs, assembled from a vast ensemble of metagenomes and including 12,556 novel candidate species-level OTUs, found no reliable evidence of novel prokaryote phylum content [30]. It may therefore seem that whatever biodiversity remains to be discovered would yield few more “major unknowns”. However, such Tree of Life reconstructions generally rely on phylogenies of conserved gene sets to identify novel clades, meaning that unknown lineages could be overlooked if their marker genes are too divergent. Moreover, across most biomes, large portions of environmental metagenomes remain taxonomically and functionally unannotated, even at permissive clustering thresholds [31], representing a significant blind spot in our grasp of the extant biological diversity on Earth. Some of these uncharacterised sequences may thus belong to genomes of unknown organisms that have so far escaped detection efforts, for instance due to accelerated evolution rates or an ancestral divergence from known organisms. Novel genes of well-characterised organisms with “open” pangenomes, divergent paralogues of known genes, and unusual mobile elements may also be expected to contribute to this “microbial dark matter” [4]. In any case, the persistence of those biological unknowns highlights the need for novel approaches complementing the current techniques to mine metagenomes for highly divergent groups.

**Fig. 1:**
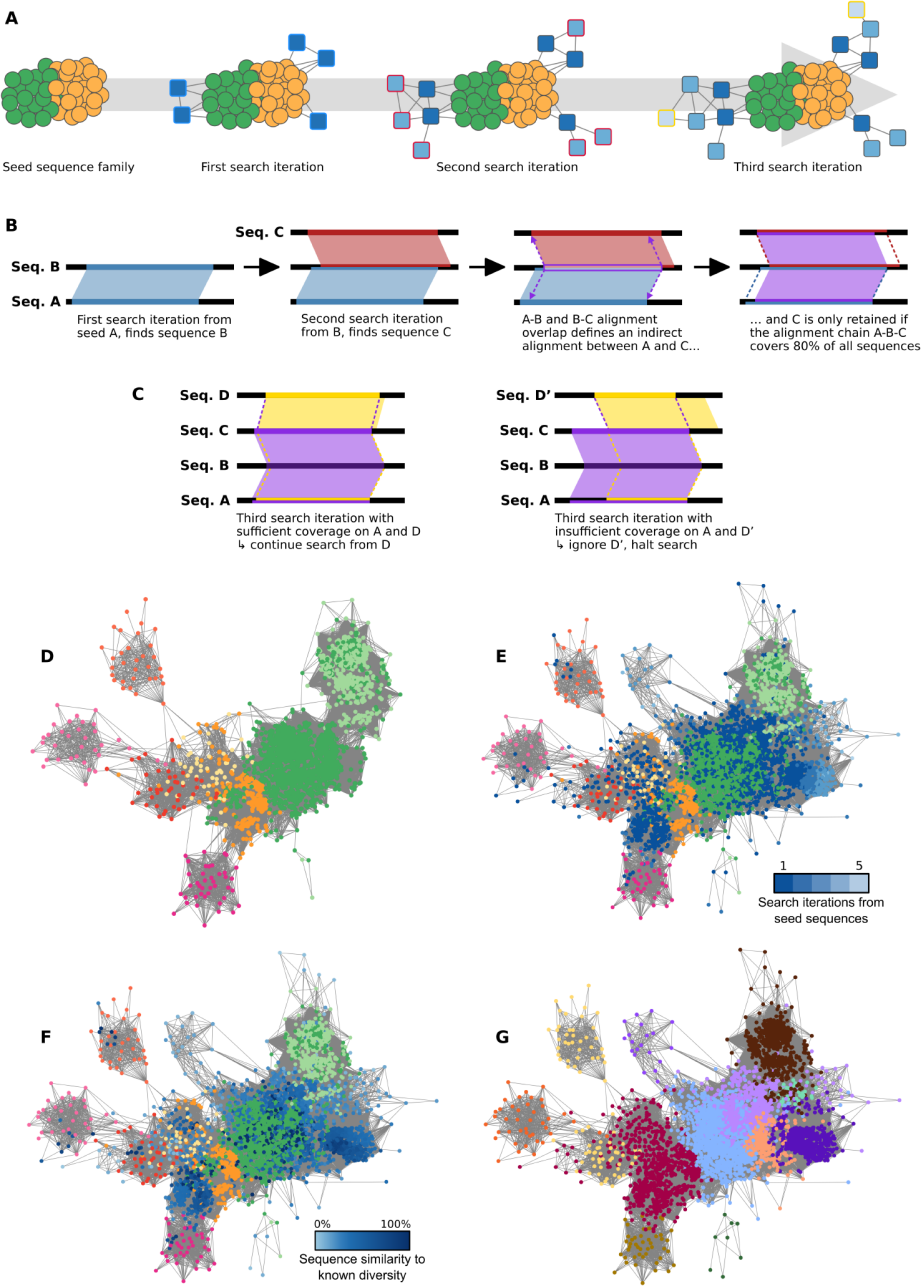
Iterative homologue search procedure. (**A**) Iterative aggregation of environmental homologues around seed sequences in a similarity network. From a set of seed sequences belonging to a given protein family (green and orange nodes), a first search iteration finds environmental homologues (dark blue nodes) for some of the seeds. A second search iteration then uses these environmental sequences as queries to find more homologues (medium blue nodes, red frame), which are themselves used as queries for a third search iteration finding further environmental homologues (light blue nodes, yellow frame). (**B**) At each iteration of the search, newly found homologues are only retained if their aligned region can be mapped back onto a seed sequence in a way that ensures > 80% coverage on all sequences along the chain of aligned sequences. (**C**) **Left**: sequence D is found after three search iterations from seed A, and its alignment with sequence C can be mapped back to A in a way that preserves 80% coverage on all sequences along the “alignment chain”. Sequence D is therefore retained and will be used as query for the next iteration of the search. **Right**: sequence D’ is found after three search iterations from seed A, but its aligned region cannot be mapped back to A without breaking the 80% coverage requirement. D’ is thus not retained as a distant homologue of A in this round of search. (**D-G**) Sequence similarity networks for SMC proteins. (D) shows seed sequences only, (E-G) show seed and environmental sequences. In (D-F), nodes representing seed sequences are coloured according to their taxonomic origin (yellow: non-DPANN archaea; orange: DPANN archaea; light green: CPR bacteria; dark green: non-CPR bacteria; shades of red: four eukaryotic SMC paralogues). In (E), environmental nodes are coloured in blue, with darker shades for sequences retrieved in earlier iterations of the search, and lighter shades for sequences retrieved later. In (F), environmental nodes are coloured in blue, with darker shades for sequences with higher similarity to the known cultured diversity, and lighter shades for sequences with less similarity. In (G), all nodes are coloured according to Louvain clusters inferred in the SSN (one arbitrary colour per cluster).

Various network-based approaches [32], in particular, have been developed to address these concerns. Sequence similarity networks, wherein pairs of primary sequences are connected according to set similarity criteria, can be employed to compare sequences from cultured and uncultured organisms [33, 34]. In 2012, Lynch et al. used sequence similarity networks to identify several candidate new lineages from environmental 16 S rRNA [35]. In 2015, Lopez et al. designed a network-based exploratory analysis to probe metagenomes for distant homologues of well-distributed gene families [36]. 86 clusters of genes broadly distributed across Domains of life were used as seeds for a two-step BLAST search inside a metagenome collection. Seed sequences were then gathered in sequence similarity networks together with their direct and indirect environmental homologues, and environmental sequences gathered in the second alignment step were more divergent from their cultured relatives than those gathered in the first round. The authors found several hundred groups of highly divergent environmental variants, some of them potentially compatible with novel major divisions of life. Consequently, (i) iterative explorations of environmental datasets allow the retrieval of increasingly divergent variants (Fig. 1A), and (ii) network-based methods are well-suited to handle this type of data, by integrating sequences with various levels of divergence within homologous gene families. This ability of similarity networks to bridge the gaps of remote homology in environmental sequence data has recently been leveraged to address “functionally unknown” aspects of microbial dark matter within the AGNOSTOS framework [37], which can help in generating biological hypotheses by mapping out, categorising and contextualising metagenome contents into different classes of increasing functional “darkness”. Sequence similarity networks have also been used in this functional context to assess how the deep-learning breakthrough in protein structure prediction may help unravelling the functionally dark regions of the natural protein space [38]. Lastly, different clustering approaches have been developed for the purpose of identifying novel protein families with no cultured representative, and which could be hosted by exclusively uncharacterised lineages [39, 40].

In this work, we conducted a more targeted exploratory search of ocean metagenomic data, aimed specifically towards identifying novel genetic diversity of well-known core gene families that could potentially belong to unknown lineages of microorganisms (including prokaryotes as well as viruses and single-cell eukaryotes). Rather than unravelling the functionally unknown regions of the sequence space, our objective is thus to investigate the increase in diversity in well-known gene families once contributions from uncultivated microbes are taken into account. Our search mined the environmental diversity of the Ocean Microbial Reference Gene Catalog (OM-RGC, version 1) dataset [41]. This extensive, non-redundant record contains sequences for over 40 million bacterial and archaeal genes, predicted from metagenomic sequencing of a large variety of marine environments across the world. At the time of initial publication, around 45% of these sequences lacked taxonomical annotation at or below the Domain level, and 43% lacked functional annotation to an eggNOG orthologous group (OG), highlighting the existence of a vast, undescribed diversity in the global oceanic microbiome, as well as the necessity of additional efforts to improve its characterisation. To perform this search, we further developed the iterative explorative strategy of environmental datasets initiated by Lopez et al. [36], by allowing distant homology searches to continue for more than two BLAST iterations, until these iterations converge and stop retrieving additional family members from a given environmental dataset. We focussed our search on highly conserved and rarely laterally transferred – hence likely ancestral – gene families. Retrieving highly divergent variants in such ancient families could indeed carry an increased biological significance, given their stability in primary sequence for many reference genomes, and could potentially guide future searches for novel putative taxonomical groups or biological functions involving these nearly universal gene families. Specifically, we used a custom dataset of 53 ancient, conserved gene families with key biological functions to initiate our iterative probing of OM-RGC (version 1). We identified highly divergent variants of multiple gene families, uncovering new putative structural and sequence variants of biologically essential proteins across taxonomical scales and various oceanic sampling sites.

## Results

### Principle of the analysis

We developed an iterative mining procedure to accumulate highly divergent environmental variants for families of genes or proteins of interest.

#### Step 1: family selection

We retained highly conserved gene families with few or no evidence of lateral gene transfers between Archaea and Bacteria (see Materials & Methods) as seeds for our research. In brief, from an initial set of nearly ten million protein sequences gathered from prokaryotic, eukaryotic, viral and plasmidic complete genomes (Table SI-1), we selected a set of 53 protein families (see Methods) with the above characteristics, potentially as old as cellular life. Most of these seed families corresponded to single protein families as defined by eggNOG, although a few of them comprised proteins from two or more closely related families. Seed families spanned a total of 125,774 sequences. Many of these families represented genes well-known to be particularly conserved and widely used for phylogenetic reconstruction and taxonomic classification, with 30 families in particular matching near-universal bacterial gene markers from the bac120 gene set with at least 50% of their sequences, including 12 families of ribosomal proteins, and seven seed families also matched archaeal gene markers from the ar53 gene set [42] (Table SI-2). On average, bacterial sequences in the selected families had 34.9% amino-acid identity to their closest archaeal homologue (and vice-versa), providing a rough empirical baseline for sequence similarity levels that could indicate Domain-level divergence in these families.

#### Step 2: retrieval of distant environmental homologues within a reference environmental database

Each selected family was used as the seed for a deep homologue-mining procedure in the OM-RGC dataset [41]. This iterative search aimed at aggregating around each seed family the diversity of its environmental homologues, including variants too divergent to produce a significant direct alignment to any seed sequence. For each family, direct oceanic homologues of seed sequences were identified in a first round of search. The OM-RGC dataset was then further queried for homologues of those homologues, and so forth until the procedure converged to find no additional environmental homologues (See Fig. 1A-E and Methods for details). We also tested the precision and recall of this approach (see Methods), to evaluate (i) how reliably our iterative procedure successfully retrieved distant homologues of seed sequences, and (ii) whether this retrieval was prone to false-positive calls, where sequences would be attained from seeds that did not share a homologous origin. In cases where fast-evolving sequences diverged up to 2.5 times faster than their slow counterparts, the search procedure was nearly systematically able to retrieve all divergent sequences (Fig. SI-1). When the evolution rate difference was four-fold, about half of the test instances successfully retrieved all divergent homologues. Finally, above a six-fold increase, seed sequences were largely unable to retrieve any divergent sequence at all. Such a pattern suggests that our method is best-suited for retrieving remote homologues that do not evolve drastically (e.g. more than four-fold) faster than the sequences used as seeds. On the other hand, the phylogenetic depth of this simulated divergence had little effect on the efficacy of retrieval, confirming that divergent variants can be used successfully to unravel further diversity in later rounds of search. Lastly, no false positive homology hit was identified over the 3402 test instances that were performed in total, i.e. homology searches only ever retrieved sequences genuinely related to the seeds. These results on simulated data show that the procedure was well-suited for finding remote homologues of conserved gene families, and safe against false-positive detection, although the higher complexity of real-world biological sequence data may be expected to yield aberrant results on occasion.

#### Step 3: assessment of the origins of the retrieved distant environmental homologues using different and broader databases

First, all the distant environmental sequences assigned to a phylogenetically conserved gene family by our iterative search were compared to known sequences present in the *nr* NCBI database by a DIAMOND search (see Methods). In addition, all retrieved environmental sequences were taxonomically assigned by a BLAST search against the OM-RGC v2 dataset (see Methods). This protocol allowed us to confirm the genetic novelty of many environmental sequences and their significant divergence in primary sequence to known and/or cultured references. Some of the retrieved environmental sequences show levels of divergence to the known diversity that are comparable with the difference between archaeal and bacterial homologues. These variants could potentially belong to uncharacterised lineages that branched away from well-known taxa long ago, although alternative hypotheses can be offered. Divergent environmental homologues could, for instance, be distant paralogues of seed sequences, that evolved faster than their known counterparts due to relaxed selective pressure after duplication, and appear environmentally conserved but not described in cultured organisms. To test this possibility, we screened the large collection of oceanic single-cell genomes GORG-Tropics [43] for co-occurrences of ‘well-known’ gene products and their highly divergent variants, which would be suggestive of paralogous origins for these variants. Likewise, bacteriophages and other viruses encode multiple key genes for cellular functions of their hosts [44, 45], sometimes resulting in clades distinct from their cellular homologues in gene phylogenies. Some environmental sequences that we identified as divergent could thus be of viral origin. To account for marine viruses in our analysis, our environmental homologues were aligned against all contigs from the GOV 2.0 viral database [46] in order to find viral assemblies encoding these variants. This third step allowed us to assign labels to each environmental sequence as (i) genetically divergent to reference known taxonomic groups, (ii) potentially member of a paralogy group, (iii) potentially viral.

#### Step 4: identification of clusters enriched in divergent environmental sequences (i.e., divergent environmental clusters)

We used the labels from Step 3 to detect clusters enriched in divergent environmental homologues in sequence similarity networks (see Methods) to identify groups of related divergent sequences that belong to environmental lineages harbouring original versions of otherwise conserved gene families.

#### Step 5: biogeographical distribution analyses

We leveraged the geographical origins of each environmental homologue of our conserved gene families (see Methods) in order to (i) distinguish oceanic samples enriched in divergent environmental homologues, which could be considered as possible hotspots of novel genetic diversity, and (ii) identify divergent clusters with broad geographical distributions (i.e., ubiquitous lineages bearing variants of otherwise conserved gene families).

### Oceanic metagenomes harbour distant homologues of highly conserved protein families

Our iterative metagenome mining procedure expanded the selected 53 seed families by a total of 826,717 environmental sequences from OM-RGC, representing just over 2% of the whole dataset. All seed families had their own set of environmental homologues, requiring an average of 7 rounds of iterative search before exhaustion. Despite metagenomic sequencing sometimes yielding shorter gene sequences than what is anticipated from genomes in culture, sequences retrieved from OM-RGC were only 4% shorter than their reference counterparts on average (Pearson *r* = 0.96, *p*-value 3.5 × 10^–30^; see Methods), suggesting that the alignment coverage constraints implemented in our search were able to filter out artificial sequences created by fragmented gene assemblies.

OM-RGC homologues of the 53 selected seed families were then aligned against proteins from the NCBI non-redundant (*nr*) database to find their closest relative in all the published sequences with taxonomically resolved annotations (Fig. 1F; Supplementary Text SI-1). Only 6.7% of all retrieved environmental sequences shared > 90% amino-acid identity with their closest characterised relative, implying that a large majority of environmental proteins cannot be accurately represented by genomes captured by current cultivation or isolation techniques. Furthermore, 20.5% of environmental variants had less than 34.9% identity with their closest *nr* relative, i.e. they diverged more from any proteins of well-characterised organisms than bacterial and archaeal homologues diverged from one another on average in the reference dataset. Environmental homologues of ribosomal protein families had generally higher similarity to their closest characterised relative than non-ribosomal environmental sequences (one-sided Kolmogorov-Smirnov test, *p*-value < 1.6 × 10^–22^; Fig. SI-2). This observation is consistent with their infrequent duplication in genomes and their specialised function within the exclusive context of the ribosomal complex, which minimises the possibility of independent function or divergent paralogues to emerge. Still, even ribosomal protein families included very divergent oceanic variants with < 34.9% identity to their closest *nr* relative (Fig. SI-2).

### Divergent sequences are broadly distributed in the ocean

**Fig. 2:**
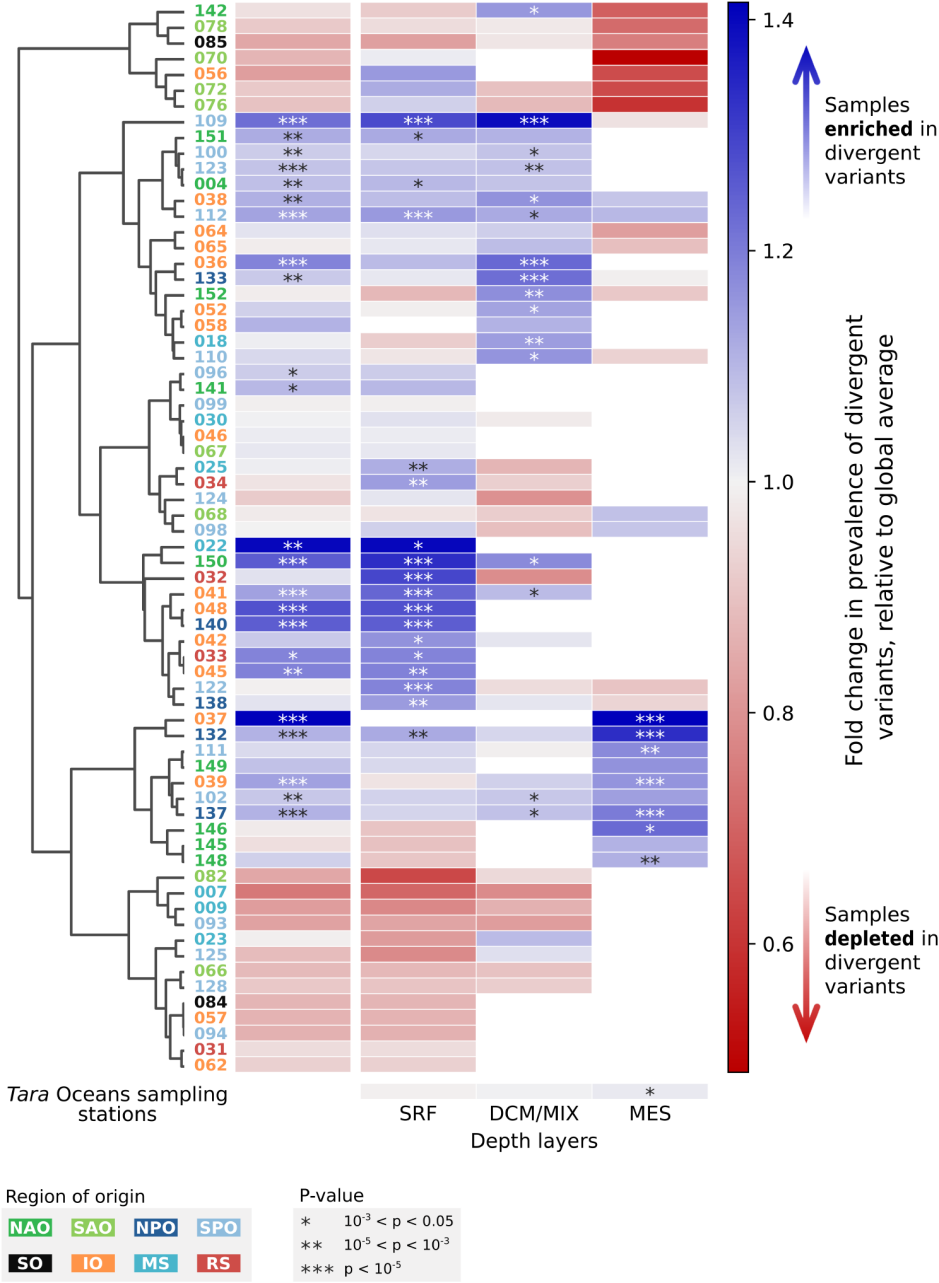
Biogeographical distribution of highly divergent environmental homologues of seed families. For each *Tara* Oceans sampling station (Y-axis) and each depth layer (X-axis), the local sample-specific fold-change in highly divergent variants (< 34.9% identity to closest *nr* relative) of 53 selected core gene families is calculated as the following ratio: % divergent variants among sequences from local sample / % divergent variants among all homologues retrieved (i.e. 20.5%). The column on the left represents the sampling station-wise enrichment in divergent variants regardless of depth. The row on the bottom represents the depth layer-wise enrichment in divergent variants regardless of sampling station. Fold-change values > 1 (blue cells) indicate a relative enrichment in divergent variants at the corresponding sample, and values < 1 (red cells) indicate relative depletion. The clustering of sampling stations based on their local enrichment profiles is represented by the dendrogram on the left. Sampling stations are numbered as in Sunagawa et al. [41], and coloured according to the sea/ocean in which they are located. Asterisks represent the significance of the local enrichment in divergent variants, using three different ranges of *p*-values from one-sided binomial tests (with Bonferroni corrections, main grid: *N* = 141; left column: *N* = 68; bottom row: *N* = 3). Abbreviations: NAO North Atlantic Ocean, SAO South Atlantic Ocean, NPO North Pacific Ocean, SPO South Pacific Ocean, SO Southern Ocean, IO Indian Ocean, MS Mediterranean Sea, RS Red Sea; SRF Surface water layer, DCM deep chlorophyll maximum layer, MIX subsurface mixed layer, MES mesopelagic layer.

Our analysis into the biogeography of these highly divergent sequences showed that niches for high discovery potential exist at all depths under the surface and in virtually all provinces of the global ocean (Fig. 2; Fig. SI-3&4). Although many samples were enriched in very divergent variants (binomial test with Bonferroni correction, see Methods), the magnitude of that effect remains moderate. The maximal rate of enrichment in divergent sequences (achieved in the mesopelagic layer sample taken at the sequencing station no. 037, in the Indian Ocean) is of + 42% relative to the global average; conversely, divergent variants still represent > 10% of all sequences even in the most depleted location (mesopelagic layer sample at sequencing station no. 070, South Atlantic Ocean). On a global scale, the distribution of divergent homologues is also remarkably uniform across oceanic layers: although mesopelagic waters are overall slightly enriched in those variants (+ 1.7% relative to global average, *p*-value 9.7 × 10^− 3^ from one-sided binomial test with Bonferroni correction of *N* = 3), many samples from surface (SRF) and sub-surface (DCM/MIX) epipelagic layers also harbour large proportions of divergent genes. All locations in the global ocean could therefore harbour prolific reserves for new microbial variants of core gene families.

### Highly divergent clusters of environmental variants expand the diversity of multiple universal protein families

Seed sequences and their (direct and indirect) oceanic homologues were gathered in family-specific sequence similarity networks (SSNs). In such networks, closely related sequences are expected to lie in close proximity, thus reflecting the structure of protein families in the network topology [47, 48]. Sequences within each SSN were therefore partitioned into network communities using Louvain clustering [49] (Fig. 1G). This higher-level view of network structures allows an easier assessment of the environmental diversity, including identifying potential sources of biological novelty in these protein families. In particular, clusters consisting exclusively or predominantly of environmental sequences (> 90% of environmental sequences), with little similarity to published sequence records (< 40% sequence identity to any non-environmental sequence in the nr database), and containing enough proteins to be unlikely the result of sequencing inaccuracies, are intuitively the most likely to correspond to genuinely novel groups of environmental homologues.

691 clusters of sequences were inferred in total across the 53 SSNs, out of which we retained 80 clusters of proteins fitting the above criteria for significant novelty potential. These 80 clusters of highly divergent sequences were distributed across nearly half (25/53) of our seed families. Remarkably, no cluster with such a high level of divergence was found in networks of ribosomal proteins, possibly due to a superior level of conservation or a higher coverage of their diversity in public sequence databases. Still, the fact that clusters of divergent environmental homologues were identified in nearly half of our selected protein families suggests that numerous key biological processes are carried out by a currently underestimated diversity of protein primary structures. In other words, environmental sequence datasets can contain sources of significant novelty even for the functionally resolved areas of the gene space [31, 37].

To assess how these groups of divergent sequences may relate to their reference counterparts, we reconstructed phylogenetic trees regrouping seed and environmental sequences from each of the 80 selected highly divergent clusters. This selection exposed an additional phylogenetic diversity in conserved protein families when environmental contributions are considered. In particular, in some families, sequences representative of certain divergent network clusters branched between or beside the main groups of archaeal and bacterial sequences. Such phylogenetic placements indicate substantial potential for novelty in the sequence space of those protein families. We detail findings of particular interest for three families in the following subsections.

### High environmental diversity in oceanic DNA polymerase clamp loaders

In a mechanism conserved across all cellular life forms, DNA polymerases process and replicate DNA by binding onto circular clamps that encircle and slide along the template DNA strand. Sliding clamps are embedded onto DNA by a pentameric clamp-loading system, which exhibits a universally conserved structure in archaea, bacteria and eukaryotes despite differences in subunit composition [50]. All clamp loaders consist of one “large” subunit (δ in bacteria, RfcL in archaea, Rfc1 in eukaryotes) complemented by four “small” subunits: three γ and one δ’ subunits in bacteria (also respectively called DnaX and HolB), four RfcS subunits in archaea, one each of Rfc 2–5 subunits in eukaryotes. All subunits are homologous to one another within and across all three Domains of life [51–54].

One of the selected seed families with divergent clusters consisted of several AAA + ATPases [55], included the clamp loader “small” subunits (CLSSUs) described above (i.e. bacterial DnaX and HolB, archaeal RfcS, and eukaryotic Rfc 2–5), as well as sequences for the bacterial replication-associated recombination protein RarA. This latter protein, present in bacteria and eukaryotes but not in archaea [56], is involved in homologous recombination and DNA repair, both in the context of DNA replication and outside [57]. The RarA protein sequence is highly conserved and also substantially homologous to DnaX, and as such was grouped alongside it in the construction of our seed families.

**Fig. 3:**
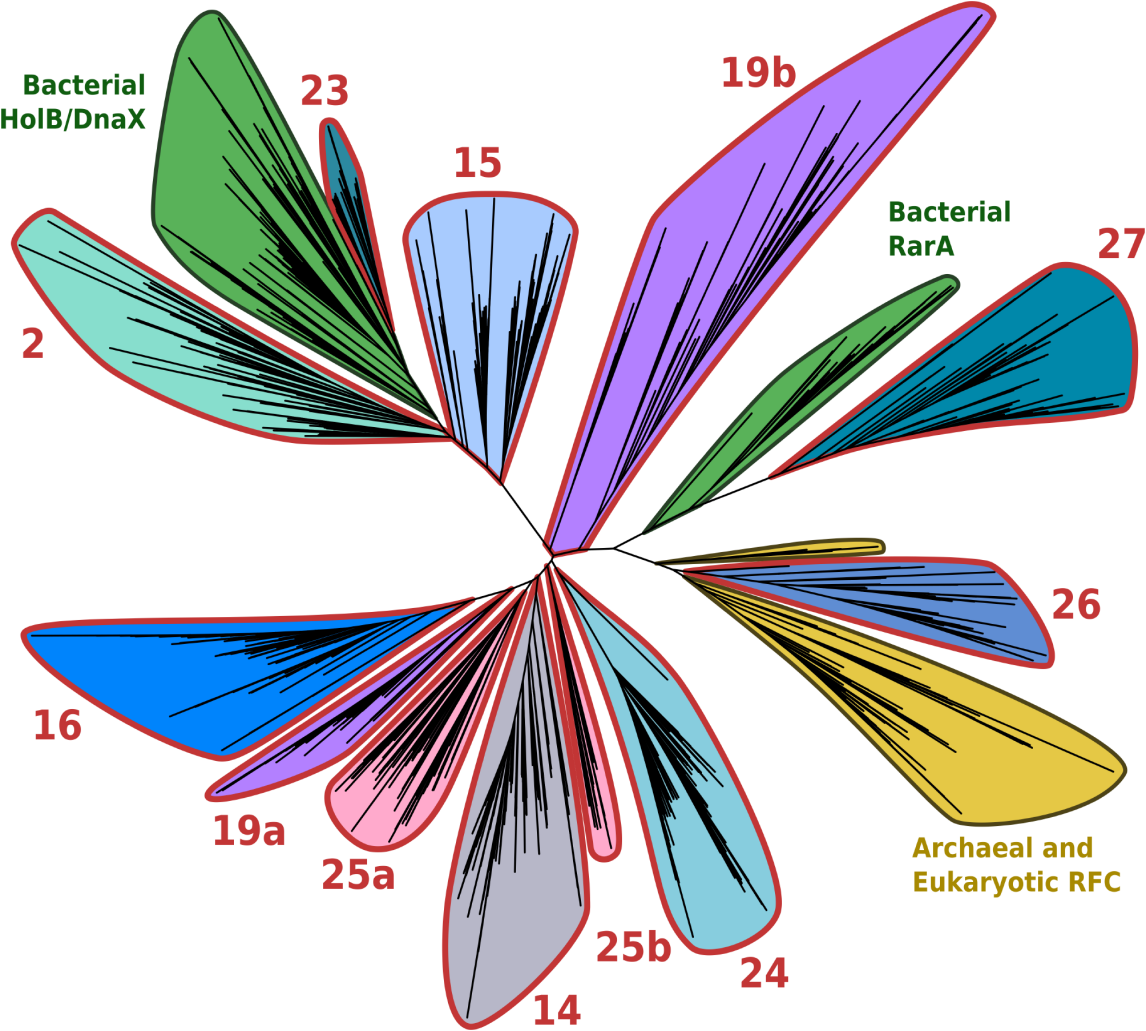
Alignment-free phylogeny of DNA clamp loader subunits HolB/DnaX/RarA/RFC and environmental homologues from significantly divergent clusters. Seed sequences are coloured according to the Domain of life of their host organism (green: Bacteria, yellow: Archaea and Eukaryotes). Groups of environmental sequences are coloured according to the network cluster they belong to in the family SSN, and outlined in red. Numerical cluster labels are inherited from Fig. SI-5 and shared with Fig. 4. Note: environmental network clusters 19 and 25 are both split into two groups in this phylogenetic tree.

The iterative retrieval of environmental homologues for this seed protein family resulted in a nearly five-fold increase of its sequence content (Table SI-2). In particular, the resulting SSN harboured 10 new clusters of highly divergent environmental homologues (Fig. SI-5). First, we analysed the phylogenetic placements of these ten divergent environmental clusters and tested the presence of paralogues and/or viral sequences within them. Owing to their high divergence in primary sequence, not all clusters translated to perfectly monophyletic groups in the phylogeny we produced (Fig. 3), though they still generally maintained some level of coherence. Among the ten environmental clusters, one had its representative sequences branch within reference archaeal and eukaryotic Rfc sequences (cluster 26), and another translated to a new clade within reference HolB/DnaX bacterial sequences (cluster 23). Additionally, one environmental cluster branched next to bacterial RarA sequences (cluster 27), and its sequences were annotated as belonging to the B subunit of the Holliday junction resolving complex RuvABC, already shown to cluster near clamp-loading proteins in sequence networks [58]. Finally, sequences from seven divergent clusters resulted in groups outside the bacterial and archaeal/eukaryotic seed sequence clans [59] in the phylogeny (clusters 2, 14, 15, 16, 19, 24, 25). eggNOG annotations for these sequences mapped them predominantly to HolB (COG0470), though it should be noted that cluster 24 contained 96% of functionally unassigned sequences.

Interestingly, taxonomic investigations into the possible origins of these divergent clusters only partly agreed with their respective phylogenetic placements. Clusters 2, 15 and 23, which appeared phylogenetically related to bacterial HolB/DnaX, were taxonomically annotated as bacterial in accordance with this placement, and clusters 2 and 15 in particular co-occurred with non-divergent HolB sequences in many GORG-Tropics single-cell bacterial genomes [43]. On the other hand, clusters 26 and 27, which lacked a taxonomic annotation, branched within reference clades respectively for archaeal and eukaryotic Rfc sequences and for bacterial RarA, yet the majority of their sequences were close homologues to bacteriophage sequences in the GOV 2.0 database [46], suggesting that clusters 26 & 27 could correspond to viral variants of CLSSUs. Another three divergent clusters (no. 14, 16 and 24), which showed phylogenetic placements away from the reference clades of either HolB/DnaX, RarA or Rfc, also seemingly represent viral genes, specifically encoded by the *Chrysochromulina ericina virus* representative of the giant NCLDV phylum. Lastly, divergent clusters 19 and 25 lacked a taxonomic annotation or representatives in bacteriophages, although cluster 19 appeared paralogous to non-divergent Rfc sequences of the archaeal TACK supergroup.

**Fig. 4:**
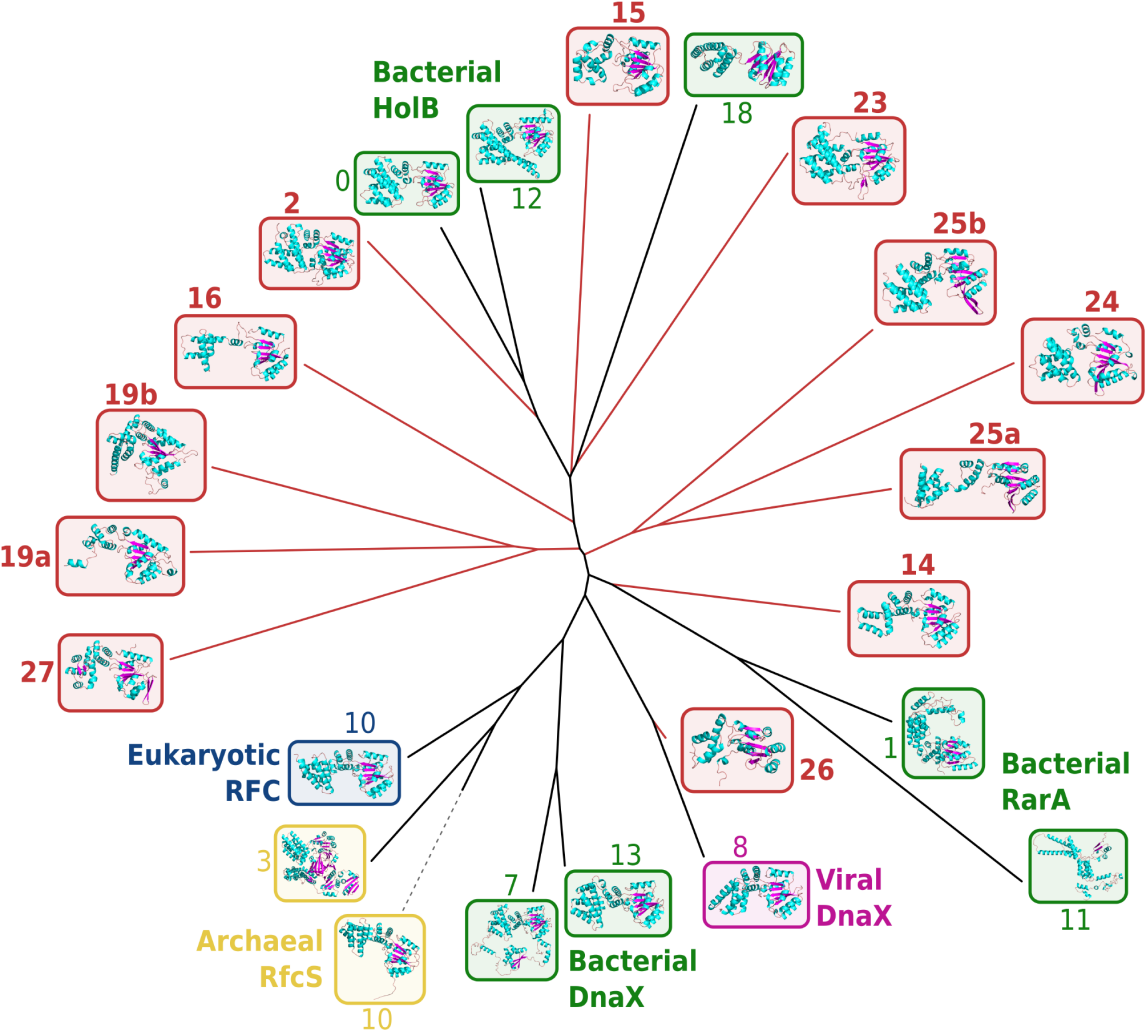
Dendrogram of tertiary structures of DNA clamp loader subunits HolB/DnaX/RarA/RFC and environmental homologues from significantly divergent clusters. Protein structures were inferred with AlphaFold and compared (all against all) using Foldseek. Leaves and structures are boxed according to the Domain of life of their host organism (green: Bacteria, yellow: Archaea, blue: Eukaryotes, magenta: Viruses). Environmental leaves and structures are boxed in red, with numerical labels corresponding to the SSN cluster they belong to, in accordance with Fig. 3 and Fig. SI-5

Lastly, we analysed the structural diversity of these divergent clusters. Protein structures were predicted for representatives of seed and divergent environmental CLSSUs using ColabFold [60, 61], and gathered in a dendrogram depicting their similarities (Fig. 4). Most seed proteins used for this comparison showed similar structures, although HolB, DnaX, RarA and archaeal/eukaryotic Rfc still formed distinct groups in the structure dendrogram. Structures inferred from environmental variants followed a pattern similar to the sequence phylogeny, with representatives from clusters 2, 15 and 23 branching near HolB references, and most other clusters translating to structures sitting outside of the main reference groups. In other words, the environmental HolB variants that we identified on the basis of primary sequence divergence also exhibited a divergence in 3D structure consistent with their phylogenetic placements.

Together, these results hint at a diversity of uncultivated marine organisms replicating DNA using various unusual proteic machineries, possibly resulting in unusual replication mechanisms operating in the ocean.

### Novel abundant clade of SMC proteins with unusual structure in actinobacteria

Another remarkable seed family consisted of SMC (*s*tructural *m*aintenance of *c*hromosomes) proteins, and we identified a small but abundant group of environmental SMC variants with strikingly singular structures within Actinobacteria.

SMC proteins are present in all Domains of life and act (as part of the SMC complex) as regulators of high-order chromosome organisation [62]. Eukaryotic genomes encode six paralogous SMC proteins (SMC1-6), due to a sequence of duplications around the time of the last eukaryotic common ancestor. Indeed, a single copy of the *smc* gene is present in nearly all archaea and bacteria, with a few exceptions. In some γ-proteobacteria a different protein complex, MukBEF, is responsible for these functions instead [63]. Bacteria from various phyla can also harbour another complex, MksBEF, alongside their SMC or MukBEF machinery [64]. MksBEF is believed to be evolutionarily related to MukBEF, and both are structurally analogous to the SMC complex, but primary sequence comparisons have ruled this structural similarity as convergent rather than due to distant homology [62]. SMC complexes are also notably absent from Thermoproteota, resulting in distinctive chromosomal dynamics and cell cycle logics [65, 66].

A typical SMC protein consists of five domains: an N-terminal domain containing a Walker A motif; a first helical chain of roughly 300 amino-acids; a central “hinge” domain; a second α-helix of comparable length to the first; a C-terminal domain containing a Walker B motif [67, 68]. This linear structure self-folds by linking the N- and C-terminal motifs into an ATPase “head”, with the two α-helix domains forming an antiparallel coiled-coil between this head and the hinge domain. This hinge then serves as a dimerisation site for a second SMC monomer, with accessory proteins binding to the ATPase heads to complete the ring-shaped SMC complex [62]. The hinge region of the SMC complex subsequently plays the essential role of mediating DNA binding, and allows the loading of SMC rings onto chromosomes [69, 70].

**Fig. 5:**
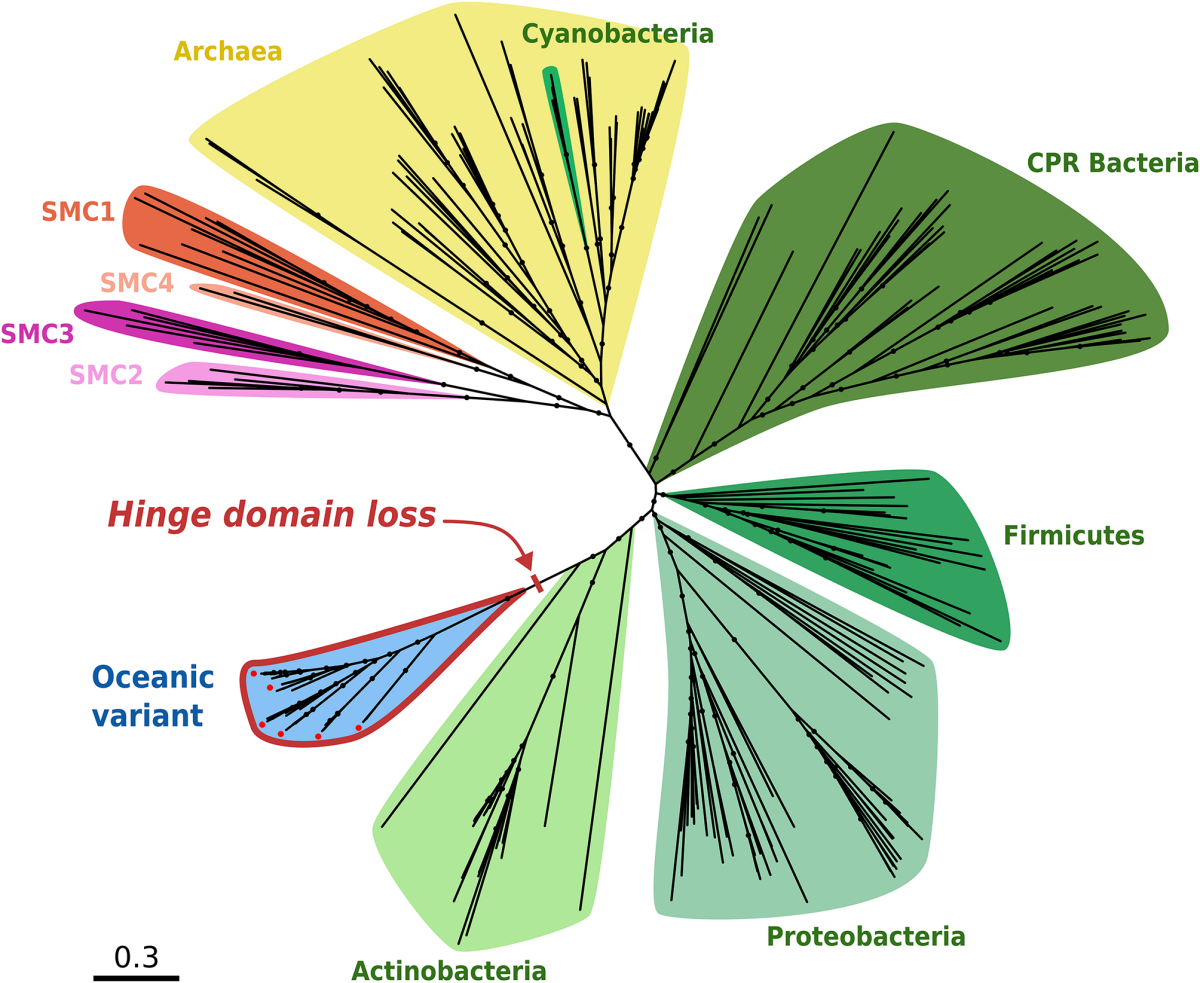
Maximum likelihood phylogenetic tree of SMC sequences and environmental homologues from significantly divergent clusters. Seed sequences are coloured according to the Domain of life of their host organism (green tones: Bacteria, yellow: Archaea, orange and purple tones: Eukaryotes). Environmental sequences are coloured in blue and outlined in red. Red dots indicate environmental sequences for which 3D structures were inferred. Black dots indicate branches with > 85% bootstrap support.

**Fig. 6:**
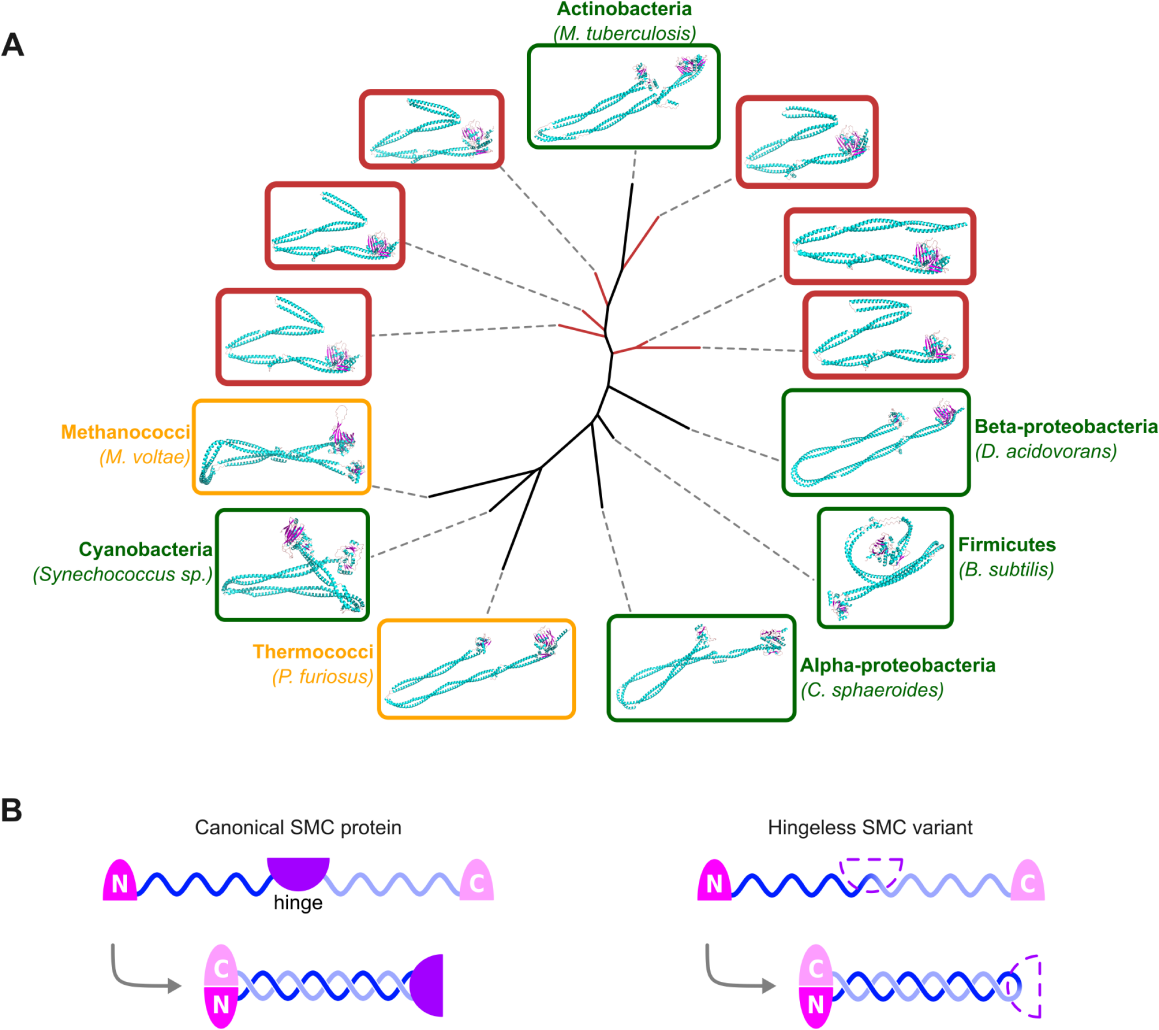
Environmental SMC homologues with divergent tertiary structure. (**A**) Dendrogram of tertiary structures of SMC sequences and selected environmental homologues from significantly divergent clusters. Protein structures were inferred with AlphaFold and compared (all against all) using Foldseek. Leaves and structures are boxed according to the Domain of life of their host organism (green: Bacteria, yellow: Archaea). Environmental leaves and structures are highlighted in red. (**B**) Schematic structure of SMC monomers. Left: canonical SMC protein with N- and C-terminal ATP-binding motifs, linked to a central hinge domain by two coiled-coil regions. This linear structure folds (grey arrow) by joining the two terminal motifs into an ATPase domain, forming a helical coiled-coil with the arm regions between the ATPase and hinge domains. Right: “hinge-less” environmental SMC homologue lacking a hinge domain. The folded protein still features the ATPase domain at one end of the coiled-coil helix, without the hinge at the opposite end

From seed sequences in this family, we retrieved a rather limited amount of environmental homologues (0.97 environmental homologue per seed sequence in this family, compared to a median value of 2.6 across all families, see Table SI-2), but one small cluster of distant environmental homologues was still identified (cluster 9 in Fig. SI-6). In the phylogeny produced from seed SMC sequences and oceanic variants from this cluster (Fig. 5), environmental sequences formed a monophyletic clade branching close to the base of seed actinobacterial sequences. These divergent environmental sequences were functionally annotated as SMC proteins (COG1196), and taxonomically as stemming from the class Actinomycetes. They were also strikingly abundant in the sequencing data, nearly seven times more so than other OM-RGC SMC homologues. Moreover, this novel oceanic clade harbours SMC-related proteins that are critically different in structure from canonical SMC proteins (Fig. 6A; average TM-score between two proteins in the divergent cluster: 0.828; average TM-score between a protein in the divergent cluster and a reference SMC protein: 0.440). Namely, these oceanic variants lack the hinge domain which is normally essential to SMC assembly and function (Fig. 6B). As such, they may be considered more similar to bacterial SbcC and archaeal and eukaryotic Rad50 proteins, thought to be distant evolutionary relatives of SMC [62]. Indeed, proteins from this ancestral family also consist of an SMC-like head and an antiparallel coiled-coil with no hinge domain, dimerising instead through a zinc-hook structure induced by a CXXC motif [71]. However, FoldSeek structural comparisons clearly discriminate between reference Rad50/SbcC proteins on one side, and SMC proteins (reference or divergent OM-RGC variants) on the other (Fig. SI-7). The zinc-hook CXXC motif conserved in Rad50/SbcC is also absent from our environmental cluster sequences, confirming them as divergent variants within the SMC diversity rather than beside it.

Several evolutionary scenarios could explain this new bacterial cluster of “hinge-less” SMC. Firstly, it could be indicative of some paralogue of SMC existing in Actinobacteria, which would then be, to the best of our knowledge, the first description of an SMC duplication in any prokaryote [72]. We view this paralogy hypothesis as rather unlikely, as hinge-less variants were not found to occur in tandem with regular SMC sequences in our screening of GORG-Tropics genomes. The hypothesis of a viral origin for these sequences is also unconvincing, as we found no homologue for these sequences in viral genomes. Alternatively, this divergent cluster could indicate the existence of an unknown lineage, supposedly branching within Actinobacteria, where the SMC hinge domain would have been lost. In any case, the substantial divergence of these environmental sequences to any gene published from a well-characterised organism, together with the loss of the essential hinge domain and their remarkably high abundance in the sampling data, suggests that we identified a new kind of biology within the SMC family. By the absence of their expected interaction site with DNA, one would speculate that these hinge-less SMC-related proteins must either perform a different function than known SMC or bind DNA through different mechanisms. The broad distribution of hinge-less SMC variants across the oceans, their monophyly and their relative abundance in the ocean microbiome suggest that they play an important, underappreciated function in this oceanic clade.

### Divergent recombinases from potentially novel groups in sub-micrometre size fractions

In a third family, consisting of RecA/RadA DNA recombinases [73], we identified other possible sources of novel diversity, including within ultra-small cell size fractions.

During the course of DNA replication, accidental double-strand breaks (DSBs) in the DNA molecule can have detrimental effects on genome stability and cell viability [74]. Recombinase proteins in the RecA/RadA family are central to homologous recombinational repair, a key replicative stress-reduction pathway that can correct DSBs as well as other types of DNA damage. This family contains the extensively studied bacterial recombinase RecA (also present in eukaryotic organelles) as well as its archaeal and eukaryotic homologues, respectively RadA and Rad51 [73, 75–77].

**Fig. 7:**
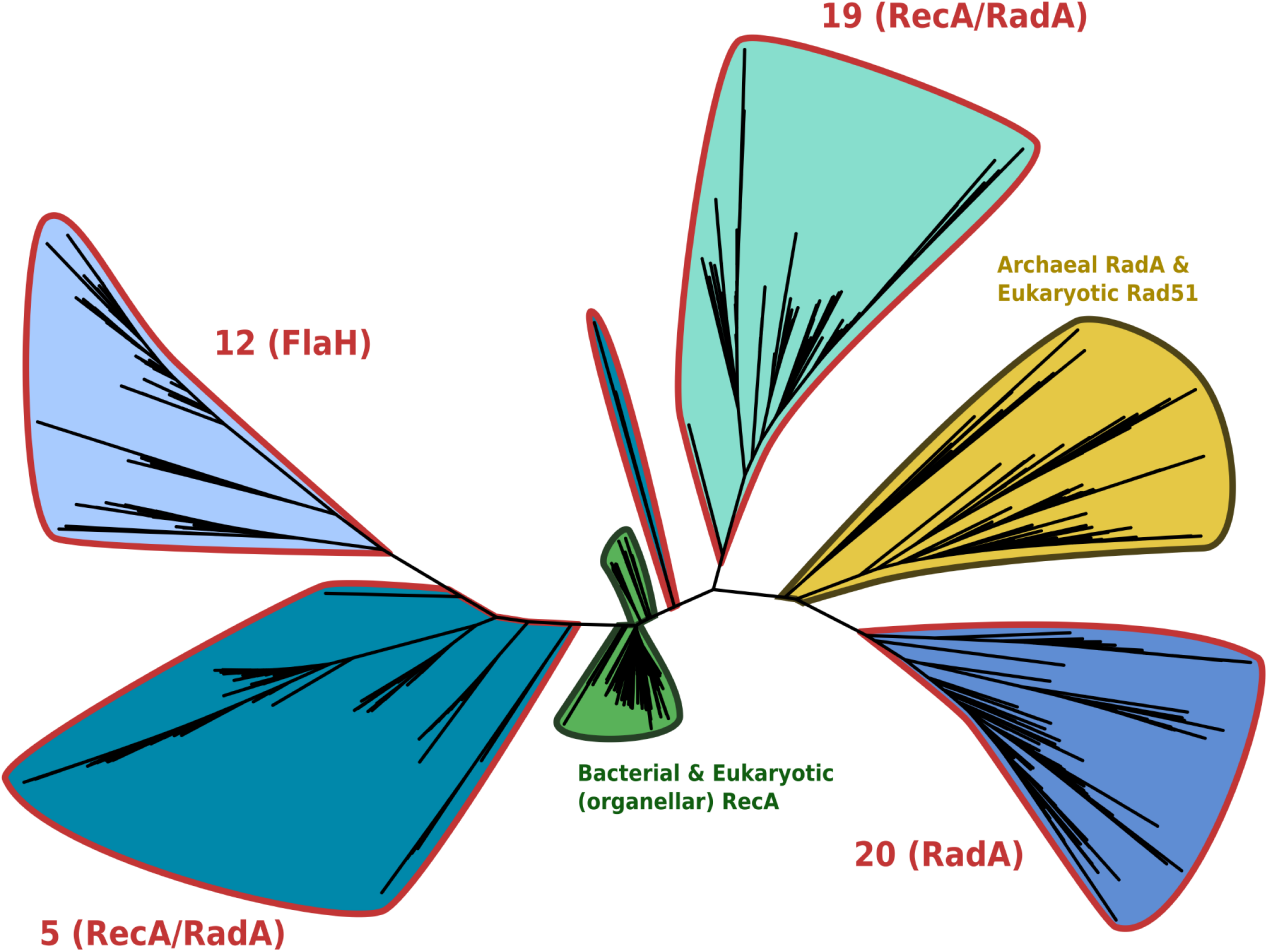
Alignment-free phylogeny of RecA/RadA sequences and environmental homologues from significantly divergent clusters. Seed sequences are coloured according to the Domain of life of their host organism (green: Bacteria and eukaryotic organelles, yellow: Archaea and eukaryotic nuclei). Groups of environmental sequences are coloured according to the network cluster they belong to in the family SSN, and outlined in red. Numerical cluster labels are inherited from Fig. SI-8

Identifying distant environmental homologues of this seed family increased its total size five-fold (Table SI-2). Within this added diversity, four clusters of environmental sequences were retained as highly divergent, totalling 1700 sequences. We analysed the phylogenetic positions of these divergent clusters, relative to the reference diversity of seed sequences (Fig. 7). In this phylogeny, sequences from a first cluster (cluster 20 in Fig. SI-8) branched near the root of archaeal seed sequences, and was functionally categorised as RadA (COG1066) in accordance with this placement. Interestingly, despite their similarity to archaeal RadA, sequences from this cluster were only found as paralogues of recombinase sequences in bacterial single-cell genomes, possibly indicating a lateral acquisition of archaeal RadA in several bacterial phyla (predominantly Cyanobacteria, Proteobacteria and Bacteroidota). A second cluster of divergent environmental sequences (cluster 12) branched within another cluster of environmental sequences (cluster 5 discussed below), seemingly in Bacteria. This cluster was predominantly annotated as ArlH (COG2874), an archaeal protein involved in the biogenesis of the archaellum, a cellular motility structure analogous to bacterial flagella [78]. Structure and sequence similarities between ArlH and bacterial RecA have previously been described [79] but, to the best of our knowledge, no evolutionary hypothesis has yet been put forth to explain this surprising homology. Still, these putative ArlH sequences were found to co-occur with recombinase genes in Euryarchaeota genomes, thus likely representing *bona fide* archaeal gene products. Finally, one cluster of distant environmental RecA homologues (COG0468) branched within bacterial sequences (cluster 5), and a final cluster, also annotated as RecA, saw its representative sequences sit between the archaeal and bacterial references (cluster 19). Both of these clusters were composed of > 50% sequences from the “ultra-small” size fraction of cells with diameters < 0.2 μm. Such cellular sizes are akin to those of CPR bacteria and DPANN archaea [80], but seed sequences from these ultra-small superphyla branched clearly within the clans of their respective Domains of life. On the other hand, > 90% of sequences from both these clusters had close homologues in GOV 2.0 assemblies, suggesting that clusters 5 and 19 correspond to RecA-like recombinases encoded in the ocean virosphere. Each of these clusters represents a sizeable phylogenetic diversity (Fig. 7) comparable to that of archaeal and eukaryotic recombinases and superior to that of reference bacterial RecA sequences, highlighting a huge genetic diversity of viral DNA recombinases that is clearly distinct from its cellular counterparts in this gene family.

## Discussion

The prevalence of biological unknowns in environmental metagenomes remains vast, such that “known unknowns” (environmental sequences, now in our databases, yet without phylogenetic proximity to reference taxa) and “unknown unknowns” (environmental sequences still waiting to be sequenced and that could turn out to be without phylogenetic proximity to reference taxa) constitute a relevant distinction to address genes, organisms, processes and interactions at play in the uncultured microbial world [31]. With our network-based, multi-marker iterative approach, we sought to understand the structure of environmental genetic variation for a range of ancient, conserved gene families with functions essential to cellular life. We found that environmental variants for those gene families could exist in marine microbiomes with considerable divergence to the known diversity, despite their “core” status and the fact that many of them have been extensively studied in the past. Moreover, these highly divergent sequences organised in (sometimes vast) cohesive groups of homology, supposedly harboured in (sometimes vast) groups of related genomes, as illustrated by the oceanic variants of DNA polymerase clamp loaders, hinge-less SMCs, and deep-branching divergent viral RecA/RadA variants.

A common issue surrounding metagenomic data is to know whether predicted genes and proteins actually exist in the sampled environment or result from aberrations in the assembly process. To avoid this pitfall, we purposefully limited our analyses to larger clusters of (similar but non-identical) sequences, from the already non-redundant OM-RGC dataset. Furthermore, the nature of our retrieval process imposes at least 80% of the length of any retrieved sequence to map back to at least 80% of a seed sequence (Fig. 1B-C). As such, recombined proteins mixing sequence fragments from several protein families are unlikely to be matched to our “canonical” seed families if exogenous regions cover more than 20% of their length. Lastly, the benchmarks we performed on simulated protein families show that sequences unrelated to the search seeds are seldom retrieved by erroneous homology calls. For these reasons, we believe that the groups of oceanic variants we discussed correspond to genuine environmental homologues of reference sequences, rather than assembly artifacts, protein recombinants, or non-homologous proteins from unrelated families.

Still, various competing scenarios of evolution and diversification can underlie highly divergent homologues such as those we detected.

First, an environmental cluster may represent deep paralogues resulting from an ancestral duplication in the gene family. Though not impossible, this hypothesis does require an explanation as to why these paralogues do not appear more broadly in the wide range of public genomes currently available, save for some unlikely event of widespread parallel gene loss across the Tree of Life. Alternatively, these divergent sequences can result from more recent gene duplications at narrower taxonomic scales, followed by a rapid divergence from their “original” copy – which, from a functional standpoint, raises interesting questions of neo- or subfunctionalisation for the novel paralogue. In polymerase clamp loader subunits, for instance, we identified a number of divergent clusters that are paralogous to more conserved homologues in Bacterial genomes. Recent experiments of directed evolution found that clamp loader complexes carried mutational potential that could compensate for changes in other polymerase components to maintain the overall fitness of the system [81]. Although the authors mainly reported results concerning point-wise compensatory mutations, the divergent variants that we observed in the HolB/DnaX family could also be balancing out impeded functions in other polymerase subunits, for instance to allow adaptation to different ecological niches. The divergent SMC proteins we identified within Actinobacteria might also be a case of gene duplication (this would then be the first description of an SMC duplication in prokaryotes), though once again it would leave unexplained why most actinobacterial genomes do not seem to carry these “hinge-less” variants. The loss of the hinge interface with DNA in this protein could correspond to the transition of an SMC complex towards a MukBEF-like mechanism, which performs comparable functions without a hinge domain [71], but the evolutionary explanations for this change, if any, remain uncertain.

Divergent homologues of highly conserved, ancestral families could also stem from uncharacterised genomes bearing these variants. Marine viruses, or other mobile elements, can be expected to encode such variants and especially those from small size fractions, such as the divergent forms of recombinase A we reported. It is possible that the divergence of these homologues could then point to radical gene changes, driven by specific selective pressures associated with non-cellular organisms. Unknown cellular lineages that diverged recently (e.g. from known genera or families) could also harbour unusual gene variants. In the functions we specifically targeted, strong constraints on sequence evolution are expected, meaning that drastic changes in intracellular processes or external selective pressure may have prompted those high levels of sequence divergence over short evolutionary timeframes. Highly divergent variants of RecA could, for instance, be the result of adaptations to different ecological niches [82]. Bacteriophages also need recombination mechanisms to invade prokaryotes, either by hijacking the host machinery or by integrating autonomous systems into phage genomes [83]. Mutations in bacterial recombinase A could therefore be providing defences against viral infection by preventing this hijacking, whereas phage-encoded recombinase variants could conversely help circumvent these defences, as well as promote phage diversification and evolution by facilitating recombination between related co-invading phages [84].

Lastly, the levels of divergence observed from some environmental groups could be compatible with novel major taxonomic groups that diverged from the established diversity some hundreds of millions, or even billions of years ago. This last hypothesis would, of course, require a lot more evidence to substantiate such a claim, and full genomes with high levels of divergence across their length would have to be produced and analysed thoroughly. Still, however remote, the possibility for new basal branches in the tree of life should not be fully discarded in the absence of conclusive evidence favouring other hypotheses.

All in all, the detection of divergent variants in key protein families, that have likely existed since cellular life began, supports the notion that major gaps remain in our knowledge of biological diversity, and that various forms of exciting new biology may be expected from unravelling this microbial world. To that end, future methodological extensions that rely less on primary sequence comparisons still appear warranted to address the whole natural diversity. The recent breakthroughs in protein structure prediction, in particular, could greatly benefit microbial dark matter analyses, as 3D structures tend to be more conserved than primary sequences during evolution. As such, the development of 3D similarity networks, connecting protein structures from cultured organisms to structures predicted from metagenomes, could offer unprecedented insights into the diversity and evolution of core gene families in natural environments, opening the door to new discoveries regarding their, possibly uncharacterised, hosts. Other types of genomic information could also prove fruitful in this regard. Gene synteny analyses, for instance, could inform biologists on evolutionary dynamics at play in microbial communities, such as recombination events and gene sharing networks [85–87], especially benefitting from the growing use of in situ microbial single-cell genomics [88, 89]. In terms of sampling, the hunt for microbial dark matter is possibly not over yet, as many sites across the global ocean still hold a reservoir of divergent environmental sequences with respect to known archaeal and bacterial sequences.

## Materials & methods

### Constitution of a conserved protein families dataset

We constituted a dataset of 9,737,821 proteins, from 4403 bacterial (including CPR), 567 archaeal (including DPANN and Asgard), 120 eukaryotic, 18,020 viral and 1586 plasmidic genomes, acquired from public NCBI databases [90] (Table SI-1). The sequence similarity network (SSN) of this protein collection was reconstructed by an all-against-all DIAMOND blastp alignment [91] (version 2.0.9, thresholds: E-value ≤ 10^− 5^, sequence identity ≥ 30%, mutual coverage ≥ 80%). This SSN contained 891,459 protein clusters (connected components). The assortative mixing [92] between Domains of life within each cluster, quantifying the preferential interconnection of sequences either with sequences from the same Domain of life, or with sequences from a different Domain, was computed using the Python package networkx [93] (version 2.8.8, function attribute_assortativity_coefficient). We retained 53 protein clusters meeting thresholds of (i) Domain assortative mixing ≥ 0.65 and (ii) 150 or more sequences from both archaea and bacteria. These 53 protein families comprised a total of 125,774 sequences.

### Mapping to reference archaeal and bacterial families in GTDB

We checked for overlaps between our selection of 53 seed families and the reference sets of conserved genes for archaea and bacteria (ar53 and bac120, respectively) maintained by GTDB [42, 94–96]. Sequences from each of our selected families were aligned against representative sequences of the ar53 and bac120 marker sets (Release 220) with DIAMOND, using thresholds of E-value ≤ 10^− 5^ and sequence identity ≥ 30% in very-sensitive mode. We then counted the number of matches between each seed family and each GTDB marker family. We retained for each seed family its closest match in the ar53 and the bac120 marker sets, defined as the marker in each set matching the highest number of seed sequences.

### Iterative retrieval of environmental homologues

We collected 40,154,822 gene sequences from the Ocean Microbial Reference Gene Catalog (OM-RGC, version 1 being the most recent version at the time our analyses were initiated) [41], alongside corresponding sampling metadata and eggNOG [97] annotations, and translated them into amino-acid sequences. An iterative search for environmental homologues in the OM-RGC dataset was conducted for the selected 53 protein families independently (building upon [36]). For each family, seed sequences were aligned against the OM-RGC protein sequences with DIAMOND (thresholds: E-value ≤ 10^− 5^, sequence identity ≥ 30%, mutual coverage ≥ 80%). Environmental sequences retrieved were used as a base for a new round of DIAMOND alignment (identical parameters) against OM-RGC. This procedure was iterated, each round using as queries the environmental sequences retrieved in the previous round, until no additional sequence was found (Fig. 1A). At each step, the aligned regions of matched sequences were checked to project back to cover > 80% of at least one seed sequence, to maintain the plausibility of distant homology between indirectly linked sequences (Fig. 1B-C). Sequences not meeting this criterion were discarded before the next search iteration. 826,717 sequences in OM-RGC were assigned to the selected protein families in this way (Table SI-2).

We calculated, for each seed family, the average length of its seed sequences on the one hand, and the average length of its environmental homologues on the other. These average values were then compared using a Pearson correlation test in order to assess whether seeds were significantly shorter or longer than their environmental counterparts.

### Precision and accuracy of our iterative retrieval protocol on simulated protein families

From a balanced binary tree with 64 leaves, we generated a collection of toy phylogenies. For each non-root node in the starting tree, new trees were created by elongating branches between the root and this node, by a factor of 1 (“null” case), 1.5, 2, 2.5, 3, 3.5, 4, 6 or 8, yielding 126 non-root nodes × 9 possible elongation factors = 1134 (non-unique) tree instances. Random sequences of 300 amino-acids were then generated and numerically evolved along the branches of these trees using pyvolve [98] (version 1.0.3, LG model). Doing three replicates per tree instance, we thus simulated a total of 3402 artificial protein families with 64 members each.

In each tree we generated, branches were only elongated from the root to one target node, and therefore only on one side of the root, leading to leaf nodes on that side being further away from the root than the leaves on the opposite side. Sequences simulated along those trees could therefore be classified as slow- or fast-evolving depending on their side in the tree. Slow-evolving sequences within the same “family” shared an average of 42.7% sequence identity. The slow-evolving subsets were each used as seeds for iterative homology searches to retrieve their own fast-evolving homologues amongst all sequences generated from all phylogenies. 3402 iterative homology searches (same parameters as for real-world data) were thus conducted, each time using the slow-evolving sequences from one simulated family to find their fast-evolving homologues within the entire set of generated sequences. The precision (percentage of true positive homology calls amongst all retrieved sequences) and recall (percentage of fast-evolving homologues successfully retrieved) of the search protocol were determined from these results, for each possible factor of divergence, and each possible depth in the tree this divergence spanned (from 1, stopping at a node directly under the root, to 6, all the way to a leaf node).

### Comparison of retrieved environmental sequences to cultured diversity

Environmental sequences retrieved for each of the 53 selected seed families were compared to published sequences from taxonomically resolved organisms in the NCBI *nr* database (downloaded in March 2020) via a DIAMOND alignment search (E-value ≤ 10^− 5^). Similarity values between environmental sequences and their closest published relative were calculated as the product of the amino-acid identity in the aligned region times the alignment coverage on the shortest sequence. Sequences were labelled ‘highly divergent’ when their identity to the closest published relative was < 34.9%, corresponding to the identity between a sequence from one prokaryotic Domain and its closest relative in the other that we observed on average in seed families.

### Biogeographical distribution of environmental gene variants

Each retrieved environmental homologue was associated with a sampling site and an oceanic depth, among the 68 locations and three depth layers (SRF, DCM/MIX, MES) listed in Sunagawa et al. [41]. The prevalence of highly divergent variants (as defined above) was estimated in each sample, i.e. each combination of location and depth layer, by performing binomial tests relative to the overall frequency of divergent sequences in all retrieved sequences, adjusted with a Bonferroni correction for multiple testing (*N* = 141 location-depth combinations with non-zero sequence count). This enrichment in highly divergent variants was also tested separately across all locations regardless of sampling depth, and across all depths regardless of sampling location (Bonferroni corrections for *N* = 68 locations and *N* = 3 depth layers, respectively).

### Sequence similarity network reconstruction and analysis

SSNs were computed for each environmentally expanded protein family by conducting all-against-all DIAMOND blastp alignments of seed and environmental sequences (E-value ≤ 10^− 5^, sequence identity ≥ 30%, mutual coverage ≥ 80%). We then inferred, using Louvain clustering (implemented in networkx, v2.8.8) [49], node communities in those networks, i.e. groups of sequences tightly connected by homology links. This clustering defined 691 communities across the 53 families in our dataset. We further selected clusters containing at least 30 sequences, of which at least 90% were from the environmental dataset, and with environmental sequences averaging 40% identity or less with their closest published counterpart. 80 such clusters, hereafter referred to as “divergent”, were identified across 25 families.

SSNs were rendered using Cytoscape (version 3.9.1) [99]. However larger networks, typically with millions of edges, made visualisations intractable. Synthetic “meta-networks” of those SSNs were created instead (Fig. SI-5, SI-6, SI-8). Rather than showing interconnections between all sequences, these represented connections between sequence clusters (as defined above): each Louvain cluster inferred in an SSN was condensed to a single “meta-node”, and two meta-nodes were linked by a “meta-edge” if the corresponding clusters were adjacent in the SSN. Meta-edges were also given a numeric weight representing the proportion of edges between clusters, relative to the total possible number of edges if the clusters had been fully connected together.

### Phylogenetic analysis of divergent clusters

Sequences from the 80 divergent clusters we identified (see above) were gathered in phylogenetic trees along with seed sequences. We used CD-HIT (90% identity threshold, version 4.8.1) [100] to dereplicate sequences from each of these 80 selected clusters, as well as seed sequences from each of the 25 corresponding families. Up to 100 sequences per environmental cluster and 200 seeds per family were selected as representatives, in order to take into account the inner diversity of each group as well as compare their phylogenetic placements relative to each other. We then computed two types of phylogenetic trees: (i) cluster-specific phylogenies, containing sequences from seeds as well as one divergent environmental cluster, inferred using maximum likelihood algorithms; (ii) family-wide phylogenies, containing sequences from seeds and all divergent environmental clusters, inferred using alignment-free techniques [101, 102].

Cluster-specific phylogenies were computed in order to assess the phylogenetic placement of each environmental divergent cluster relative to the known diversity (represented by seed sequences). Sequences from each divergent cluster were aligned with corresponding seed sequences using Mafft (version 7.520, 1000 iterative refinement cycles) [103]. These alignments were then trimmed using trimAl (version 1.4.1) [104], and phylogenies were produced using IQ-TREE (version 1.6.12, 1000 bootstrap replicates) [105–107].

Next, family-wide phylogenies, grouping together seed sequences and all divergent clusters from each family, were computed to represent the overall genetic diversity of each family when environmental variants are taken into account [101, 102]. *k*-mer-based distance matrices were computed between all representative sequences of a family using jD2Stat (version 1.0, *k* = 7) [108], and used to infer Neighbour-Joining trees with RapidNJ (version 2.3.2) [109]. All trees were rendered and annotated in iTOL (version 6.9) [110].

### Taxonomic annotation of environmental sequence clusters

We assessed the taxonomic distribution of environmental variants in our SSN clusters, using annotations from both versions of OM-RGC. Environmental sequences retrieved by our search were aligned against the updated OM-RGC v2 dataset (which includes sequences from v1) [111], using blastn with thresholds of E-value ≤ 10^− 5^ and nucleotide identity ≥ 90% with a bidirectional alignment coverage of ≥ 80%, to identify their corresponding sequences in the updated dataset. Each sequence was then assigned a taxonomic lineage, based on either its classification in OM-RGC v1 or the classification its corresponding sequence in v2, depending on which annotation had the better taxonomic resolution. Then, for each environmental cluster, we applied a majority rule to identify the taxonomic group with the finest resolution that was represented in > 50% of sequences within that cluster.

### Identification of viral homologues for environmental variants

To assess whether environmental homologues identified in our search could correspond to viral gene products, we compared all retrieved sequences to viral assemblies from the GOV 2.0 database (version dated 24 April 2019) [46]. Nucleotide sequences of our marine variants were thus aligned against all GOV 2.0 viral contigs of length > 5 kb using blastn (E-value ≤ 10^− 5^, nucleotide identity ≥ 30%, query cover ≥ 80%). After applying these blastn filters, all remaining query sequences (i.e. environmental homologues retrieved in our search) had ≥ 68.9% nucleotide identity to their best viral match. For each sequence cluster in our similarity networks, we then counted environmental sequences matching viral assemblies with a two-tier approach, accounting for sequences either directly encoded in viral genomes (≥ 95% nucleotide identity) or otherwise homologous to viral sequences.

### Determination of paralogy relationships

We sought to test whether some clusters of environmental variants could represent divergent paralogous groups, co-encoded with less divergent copies of the same gene. To that end, we aligned all retrieved environmental homologues to single-cell prokaryote genomes from the GORG-Tropics database [43] (version dated 24 October 2024) with blastn (applying threshold of E-value ≤ 10^− 5^). Sequences were considered to be encoded in these single-cell genomes if they aligned to a contig with at least 90% nucleotide identity over at least 95% of their length. Two clusters of environmental sequences in the same sequence similarity network were then considered as representing paralogy pairs if sequences from both clusters co-occurred in at least 10 GORG-Tropics genomes, either on different contigs or on non-overlapping regions of the same contig.

### Inference and comparison of protein tertiary structures

3D structures were inferred for a selection of representative sequences in the SSNs of SMC proteins and DNA clamp-loading subunits.

For clamp loaders, one sequence was selected as representative for each cluster in the SSN. Divergent environmental clusters were represented by the environmental sequence with the highest degree (number of edges in the SSN) to other environmental sequences within the cluster; other clusters were represented by the reference sequence with the highest degree to other references in the cluster. For SMC proteins, which have a significantly longer primary sequence (around 1200 amino-acids), we sought to reduce the number of structures to infer *de novo.* Six sequences from the divergent cluster of environmental SMC variants were chosen arbitrarily (all had maximal degree, because the cluster was fully connected), and public AlphaFold structures [61, 112] were acquired from UniProt [113] to represent reference SMC sequences (UniProtKB accessions: P9WGF2, Q5N0D2, A3PMS2, A9BZW2, P51834, Q69GZ5, Q8TZY2) and their Rad50/SbcC homologues (UniProtKB accessions: A0A7I7YPX7, A5GLL1, O68032, A0A210VWK9, A0A640H0H1, P62134, P58301).

Structures were inferred for selected clamp loaders and environmental SMC sequences using ColabFold (v1.5.2, default parameters) [60]. Then, reference and environmental clamp loader structures were compared using FoldSeek (version 7-04e0ec8, all-against-all, easy-search mode, no pre-filter, alignment by TM-Align) [114]. Inferred environmental SMC structures were compared with UniProt reference SMC structures following the same protocol. For both protein families, these comparisons were used to construct dendrograms with RapidNJ [109], taking as distance metric between two structures the average local distance difference test (lDDT) score of the corresponding bidirectional structural alignment [115]. Dendrograms were plotted in iTOL [110] and annotated with 3D models of the protein structures rendered by PyMOL (version 2.5.5) [116].

## Electronic supplementary material

Below is the link to the electronic supplementary material.

Supplementary Material 1

Supplementary Material 2

Supplementary Material 3

Supplementary Material 4

## Data Availability

The OM-RGC dataset analysed in this study is available from this webpage: http://ocean-microbiome.embl.de/companion.html. The dataset of conserved protein families constructed for this analysis is available from this Figshare project: https://doi.org/10.6084/m9.figshare.24893910.v1. Source code for the iterative retrieval of environmental homologues can be found on the following repository: https://github.com/TeamAIRE/SHIFT.
